# NALCN expression is down-regulated and associated with immune infiltration in gastric cancer

**DOI:** 10.3389/fimmu.2025.1512107

**Published:** 2025-02-12

**Authors:** Xuewei Li, Na Wu, Chen Wang, Beibei Pei, Xiaoyan Ma, Jun Xie, Wenhui Yang

**Affiliations:** ^1^ Department of Biochemistry and Molecular Biology, Shanxi Key Laboratory of Birth Defect and Cell Regeneration, Shanxi Medical University, Taiyuan, China; ^2^ MOE Key Laboratory of Coal Environmental Pathogenicity and Prevention, Shanxi Medical University, Taiyuan, China; ^3^ Department of Digestive Oncology, Cancer Center, Shanxi Bethune Hospital, Shanxi Academy of Medical Sciences, Tongji Shanxi Hospital, Third Hospital of Shanxi Medical University, Taiyuan, China; ^4^ Department of Gastroenterology, Shanxi Province Cancer Hospital/Shanxi Hospital Affiliated to Cancer Hospital, Chinese Academy of Medical Sciences/Cancer Hospital Affiliated to Shanxi Medical University, Taiyuan, China

**Keywords:** NALCN, gastric cancer (GC), immune infiltration, tumor immune microenvironment (TIME), biomarker

## Abstract

**Background:**

NALCN has been identified as a tumor suppressor gene, and its role in human cancer progression has garnered significant attention. However, there is a paucity of experimental studies specifically addressing the relationship between NALCN and immune cell infiltration in gastric cancer (GC).

**Methods:**

The expression levels of NALCN in tumor tissues, peripheral blood and gastric cancer cells lines from patients with GC were assessed using RNA sequencing, immunohistochemistry (IHC) staining and RT-qPCR. Data obtained from the Gene Expression Omnibus (GEO) and The Cancer Genome Atlas (TCGA) databases were utilized to investigate the correlation between NALCN expression and immune cell infiltration in GC. Subsequently, the relationship between NALCN expression and infiltrating immune cells in GC tissues was examined through immunofluorescence method. Additionally, *in vitro* experiments were conducted to evaluate the impact of NALCN knockdown on T cells function in GC cell lines.

**Results:**

RNA sequencing analysis revealed that NALCN expression was significantly downregulated in GC tissues. Specifically, NALCN levels were lower in GC tumor tissues and plasma compared to adjacent non-tumor tissues and healthy controls. Consistent with these findings, the expression trend of NALCN mRNA in the GEO database mirrored the experimental results. Mechanistically, NALCN knockdown markedly enhanced cell proliferation, colony formation and migration while reducing apoptosis rates in AGS and GES-1 cells. Analysis of the TCGA database indicated a positive correlation between NALCN expression and the infiltration of B cells, cytotoxic cells, immature dendritic cells (iDC) cells, CD8^+^ T cells, and others in GC tissue. Conversely, Th17 and Th2 cells infiltration exhibited a negative correlation with NALCN expression. Immunofluorescence staining confirmed that B cells and CD8 T cells were more abundant in GC tumor tissues with high NALCN expression, whereas Th17 and Th2 cells were less prevalent. Subsequently, we co-cultured GC cells transfected with NALCN knockdown or control vectors along with their supernatants with T cells. The results demonstrated that NALCN knockdown in GC cells or their supernatants inhibited T cell proliferation compared to control conditions. Moreover, NALCN may play a role in glucose and glutamine uptake.

**Conclusions:**

NALCN facilitates immune cell aggregation in GC and has potential as a biomarker for immune infiltration.

## Introduction

Gastric cancer (GC) is one of the most prevalent malignancies of the digestive tract globally. According to Global Cancer Statistics 2022, it ranks fifth in incidence and is the fifth leading cause of cancer-related mortality, imposing a substantial burden on public health (1). Although both the incidence and mortality rates of GC have shown a gradual decline, the global disease burden remains significant, with the highest incidence observed in East Asia and Eastern Europe, and the highest mortality rates in East Asia (2). Currently, surgical resection remains the primary treatment for early-stage GC patients and is the only curative option available. However, only a limited proportion of GC patients are eligible for radical surgery. Despite notable advancements in diagnostic and treatment approaches, the overall cure and survival rates for GC remain disappointingly low, and long-term overall prognosis to be poor. This underscores the urgent need to address the considerable disease burden posed by GC (1, 3).

Currently, immunotherapy has made achieved substantial advancements relative to traditional systemic anti-tumor therapies, including chemotherapy, radiotherapy, and targeted therapies, thereby significantly enhancing progression-free survival and overall survival in patients with GC (4, 5). Previous studies have demonstrated that immunotherapies modalities such as immune checkpoint inhibitors (ICIs), adoptive cell therapy, cancer vaccines, and chimeric antigen receptor (CAR) T cell therapy have been extensively applied in the treatment of advanced GC (6–8). However, there is growing evidence that most patients do not benefit from immunoantitumor therapy such as ICIs, and some patients benefit briefly from immunotherapy and then progress rapidly (9). This phenomenon may be associated with the tumor immune microenvironment (TIME). Consequently, to identify novel immunotherapeutic targets for GC, screening for specific immune-related molecules has become a clinical priority.

NALCN is a relatively new cation channel discovered in recent years (10), which is mainly a group of genes associated with nerve. NALCN was first discovered in rat brains (11) and is extensively expressed in neurons across the central nervous system of both mammals and invertebrates (12–14). NALCN channels modulate the resting membrane potential of neurons by facilitating sodium leak currents, thereby playing a crucial role in various biological processes, including nerve excitability, circadian rhythm regulation, and respiratory rhythm modulation (15–17). Research has demonstrated that functional mutations in NALCN are associated with the development of neurological disorders (18).

Recently, results from Richard J. Gilbertson’s team have shed new light on NALCN. They discovered that the transport of epithelial cells to distant tissues is modulated by NALCN, thereby identifying NALCN as a critical regulator in cancer metastasis (19). Chen et al. found that NALCN is a key gene for malignant mutations in normal human liver cell lines (20). However, the potential mechanisms by which NALCN influences tumor development and its immune interactions with GC remain to be elucidated. Additionally, experimental studies investigating the association between NALCN and immune cell infiltration are currently limited.

In this study, we initially identified differentially expressed mRNAs in 22 paired GC tissues and their corresponding adjacent non-tumor tissues using RNA sequencing. Our results indicated that NALCN expression was significantly lower in GC tissues compared to adjacent non-tumor specimens. Subsequently, we validated the expression levels of NALCN in a large cohort by expanding the sample size and incorporating the data from the Gene Expression Omnibus (GEO). Furthermore, we investigated the association of NALCN with immune cells in the TIME, which was further supported by *in vitro* experiments and comprehensive database analyses.

The objective of this study was to elucidate the critical role of NALCN in GC progression and to characterize immune cell infiltration within the tumor microenvironment. Furthermore, further revealing the potential relationship between NALCN and tumor infiltrating immune cells, as well as the mechanism by which NALCN influences this process. Ultimately, identifying new therapeutic targets for GC is expected to fundamentally transform the current treatment landscape.

## Materials and methods

### Patient samples and ethics statement

Fresh frozen GC tissue, adjacent non-cancerous tissue and paraffin-embedded tissue blocks were collected from 22 pairs of surgically resected specimens obtained between 2019 and 2023 from Shanxi Cancer Hospital (Shanxi, China). Additionally, peripheral blood samples were collected from 91 newly diagnosed GC patients who had not undergone surgery, radiotherapy, chemotherapy or immunotherapy between 2023 and 2024, as well as from 69 healthy volunteers. This study adheres to the ethical principles outlined in Declaration of Helsinki and has been approved by the Ethics Committee of Shanxi Medical University. All GC diagnoses were confirmed by histopathological examination. Written informed consent was obtained from all participants or their authorized representatives.

### RNA sequencing assay

For mRNAs sequencing, RNA was isolated with Trizol method from 22 gastric tumor tissue and corresponding adjacent noncancerous tissues, and purified with RNease Mini kit. The Illumina platform (San Diego, CA) was utilized for RNA sequencing. After quality control, the processed sequencing data were aligned to the reference genome. Gene-mapped reads were quantified to determine gene expression levels. Differential expression analysis was performed using DESeq2, and the results were visualized. To identify differentially expressed genes (DEGs), we applied filtering criteria of p-value < 0.05 and an absolute fold change |FC| > 2, thereby distinguishing both upregulated and downregulated genes.

### Database

We retrieved two gastric cancer data sets, GSE66229, GSE65801, from the GEO database (https://www.ncbi.nlm.nih.gov/geo/) to analyzed NALCN expression in GC and adjacent non-tumor tissues. Additionally, we obtained NALCN data from gastric cancer samples in the TCGA database (https://www.cancer.gov/ccg/research/genome-sequencing/tcga). Immune cell infiltration scores for these samples were estimated using single-sample gene set enrichment analysis (ssGSEA) based on gene expression profiles. Subsequently, we investigated the correlation between NALCN expression and immune cell infiltration.

### Real-time quantitative PCR

Total RNA was extracted from the peripheral blood of GC patients using the RNAiso Blood reagent (TaKaRa 9112) in accordance with the manufacturer’s instructions. From cells samples, total RNA extraction was performed using the TaKaRa MiniBEST Universal RNA Extraction Kit (TaKaRa 9767), following the manufacturer’s protocol. The concentration and purity of RNA were determined using a NanoDrop spectrophotometer. Subsequently, 0.05-1ug of total RNA was reverse- transcribed into cDNA using the MonScriptTM RTII ALL-in-One Mix with dsDNase (Monad, MR05101). RT-qPCR was conducted using TB Green^®^ Premix Ex TaqTM II (Takara, RR820A) on a QuantStudio real-time PCR system (Invitrogen). Genes expression levels were quantified using the 2^-ΔΔCt^ method. All primer sequences were designed and synthesized by Sangon Biotech (Shanghai, China) and are listed in **Table 1**.

**Table 1:** Specific primers sequences used in RT-qPCR.

Gene	Forward primer (5’-3’)	Reverse primer (5’-3’)
GAPDH	GGAGCGAGATCCCTCCAAAAT	GGCTGTTGTCATACTTCTCATGG
NALCN	CAGAAACTTTTGCCGGGTGG	CACTTGGCCAGTTTTCCAGC
PD-L1	TGACCTACTGGCATTTGCTGAACG	CACTGCTTGTCCAGATGACTTCGG

### Immunohistochemistry

Tissue sections were cut to a thickness of 4 μm. Tumor slides were subjected to deparaffinization and hydration. Antigen retrieval was performed using sodium citrate buffer (PH6.0). Endogenous peroxidase (Zhongshan Jinqiao PV-6000) was blocked by 3% H_2_O_2_ at room temperature for 10 minutes. Primary antibody NALCN (1:20 dilution, Novus, NBP1-90027) was incubated overnight at 4°C. On the second day, the secondary antibody (Zhongshan Jinqiao PV-6000) was incubated at room temperature for 30 minutes. Incubate at BAD room temperature for 2-3 minutes for color development, redye with hematoxylin for 1-2 minutes, and rinse. Brine differentiation, ammonia back blue for a few seconds. Dehydrated, neutral resin seal. Look under the microscope and take pictures. The scoring methodology incorporated a thorough evaluation of both staining intensity (graded on a scale of 0 to 3) and the percentage of positively stained cells (ranging from 0 to 100%). These two parameters were subsequently multiplied to generate an integrated IHC score, which serves as a quantitative indicator of protein expression levels.

### Cell line and cell culture

Human GC AGS cells were cultured in F12K medium (Sigma, N3520), while human GC HGC27 cells, MKN45 cells and immortalized human gastric epithelial cells GES-1 cells were maintained in RPMI-1640 medium (Gibco). All cell cultures were supplemented with 10% fetal bovine serum (FBS, Gibco) and incubated at 37°C in a humidified atmosphere containing 5% CO_2_. Routine mycoplasma contamination tests were performed using the MycoAlert Mycoplasma Detection Kit (Lonza), ensuring all cell cultures remained mycoplasma-free.

### Cell transfection

Small interfering RNA (siRNA) specific to NALCN (designated as siNALCN#1, siNALCN#2, siNALCN#3) and negative controls siRNA (siCtrl) were synthesized by GenePharma (Shanghai). Cells were seeded in 6-well plates and maintained in F12K medium with 10% FBS at 37°C under a 5% CO_2_ atmosphere. Following the manufacturer’s protocol for Lipo8000 transfection reagent (Bryotime, C0533), on the next day when cells density reaches 70-80%, siNALCN was complexed with Lipo8000 in serum-free medium, gently mixed, and incubated at room temperature for 20 minutes before being added to the 6-well plates, The cells were then cultured at 37°C and 5% CO_2_ for 48 hours. RT-qPCR was employed to assess the knockdown efficiency of target genes. The sequences utilized are detailed in **Table 2**.

**Table 2:** The siRNA sequences.

Gene	Sense (5’-3’)	Antisense (5’-3’)
siNALCN#1	GGAAUGUAACCUGGAAUAGUUdTdT	AACUAUUCCAGGUUACAUUCCdTdT
siNALCN#2	CACCCGUGGUUGCCAUCUAUUdTdT	AAUAGAUGGCAACCACGGGUGdTdT
siNALCN#3	GCACGCUUCAACGCAUCUAAAdTdT	UUUAGAUGCGUUGAAGCGUGCdTdT
siCtrl	UUCUCCGAACGUGUCACGUdTdT	ACGUGACACGUUCGGAGAAdTdT

### Western blot

Total protein was extracted from cells using RIPA lysate buffer supplemented with a protease inhibitor cocktail tablet and phosphatase inhibitors (Roche, USA). The protein concentration was quantified by BCA protein detection kit (Beyotime, China, P0012S). Protein samples were resolved by 10% SDS-PAGE and subsequently transferred onto PVDF membrane via electrophoretic transfer. The membranes were blocked with 5% skim milk in Tris-buffered saline with Tween 20 (TBST) for 2 hours at room temperature. Primary antibodies used were anti-NALCN (Novus, NBP 2-22410), anti-PD-L1 (proteintech, 66248-1-Ig), and anti-cyclin D1 (Wanleibio, WL01435a) and anti-GAPDH (proteintech, 60004-1-Ig), which were incubated overnight at 4°C. On the following day, the membranes were incubated with the corresponding secondary antibodies for 2 hours at room temperature. Finally, the membranes were developed using the Chemiluminescent Substrate Reagent Kit (Invitrogen).

### Cell Counting Kit 8

Cell viability was assessed using the Cell Counting Kit 8 (CCK-8 kit, Biyuntian Biotechnology, C0037) in accordance with the manufacturer’s instructions. Approximately 2×10^3^ cells were seeded in each well of a 96-well plate and cultured for various time points (24, 48, 72 and 96 hours). Subsequently, 10 μL of CCK-8 solution was added to each well, followed by an additional incubation period of 1 hour. The absorbance was then measured at 450 nm.

### Colony formation assay

A total of 1×10^3^ AGS, GES-1 cells were seeded in 6-well plates. After 14 days, when visible colonies formed, the cells were gently washed 2-3 times with PBS and fixed with 4% paraformaldehyde solution (Biosharp, BL539A). Following the removal of the fixative, the cells were washed 3 times with PBS. Staining was performed using 0.5% crystal violet solution (Solarbio, China), after which the staining solution was discarded and the cells were rinsed with PBS. Finally, the number of colonies in each well was counted.

### Transwell assay

Transwell assay was employed to investigate cell migration. A serum-free medium containing a cell suspension was seeded into the upper chamber, which featured a polycarbonate microporous membrane (LABSEKLECT, 14341). The lower chamber was filled with medium supplemented with 10% FBS. After incubation at 37°C and 5% CO_2_ for 24 hours, cells remaining in the upper chamber were gently removed using cotton swabs. Migrated cells were then fixed with 4% paraformaldehyde and stained with 0.5% crystal violet (Solarbio, China). Three random fields were selected under an inverted microscope for observation, imaging, and cell counting.

### Cell cycle analysis

The AGS and GES-1 cells were resuspended with 1 mL of PBS and gently mixed with 3 mL of absolute ethanol by vortexing. Subsequently, the cells were fixed overnight at 4°C. On the following day, the cells were centrifuged at 500g for 10 minutes. The supernatant was carefully discarded, and the cell pellet was resuspended in PBS and incubated for 10 minutes. After a second centrifugation under the same conditions, the supernatant was again discarded, and the cells were resuspended in the DNA staining solution (Yeasen), and incubated in the dark at 25°C for 30 minutes. Finally, cell cycle distribution was using flow cytometer.

### Cell apoptosis analysis

Apoptosis was assessed using the Annexin V-fluorescein isothiocyanate (FITC) apoptosis detection kit (BD Biosciences Pharmingen, 556547) following the manufacturer’s instructions. Cells were harvested, washed with PBS, resuspended in binding buffer, and stained with annexin V-FITC and propyidium iodide under dark conditions for 15 minutes. Following staining, cells were washed again to remove unbound dye. Apoptotic cells were analyzed by flow cytometry (BD Biosciences), and the data were processed using FlowJo version10.6.1 software (TreeStar).

### Immunofluorescence

Tissue sections were cut to a thickness of 4 μm. Slides containing tumors samples were deparaffinized and rehydrated through a graded alcohol series. Permeabilization was performed using 0.3% TritonX-100 at room temperature, followed by antigenic retrieval with either sodium citrate buffer (PH 6.0) or EDTA solution (PH 9.0). Sections were blocked with 0.5% goat serum and incubated overnight at 4°C with the primary antibodies. On the following day, slides were incubated with the secondary antibody for 30 minutes at room temperature. Nuclei were counterstained with DAPI, and images were captured using a fluorescence microscope. The primary antibodies used were as follows: CD8 (1:200 dilution, proteintech, 66868-1-Ig), CD4 (1:400 dilution, proteintech, 67786-1-Ig), IL-4 (1:100 dilution, Abclonal, A5649), IL-7A (1:100 dilution, proteintech, 26163-1-AP), CD20 (1:400 dilution, proteintech, 60271-1-Ig). The secondary antibodies used Goat Anti-Mouse IgG (1:200 dilution, Boster, BA1126) and Goat Anti-Rabbit IgG (1:200 dilution, Boster, BA1142).

### T cells activation and expansion

Peripheral blood mononuclear cells (PBMCs) were isolated from the peripheral blood of healthy volunteers. T cells were subsequently purified using the Pan T Cell Isolation Kit, human (Miltenyi Biotec,130-096-535), following the manufacturer’s protocol. CD3/CD28 magnetic beads (30 μL) were resuspended with 1 mL of complete culture medium and placed on a magnetic rack for 1 minute to separate the beads. The supernatant was carefully aspirated, and the T cells were resuspended in an appropriate volume of complete medium, adjusting the concentration to 1×10^6^/mL. The magnetic beads were then added to 6 mL T cell suspension, mixed thoroughly, and transferred to a 24-well plate with 2 mL per well. Culturing was performed at 37°C with 5% CO_2_. Activated T cells were subsequently co-cultured with AGS cells or AGS cells treated with siNALCN, as well as their respective supernatants. On days 4 and 6, the expression levels of CD107a, Ki67 and granzyme B in T cells, along with T cells proliferation and apoptosis, were detected.

### Flow cytometry

A single-cell suspension was generated through mechanical dissociation. Flow cytometry analysis was conducted on BD LSR II and BD FACS Symphony instruments, following the manufacturer’s protocols. Cells sorting was performed using either a BD Aria II or MoFlo Astrios EQ sorter. Flow cytometry data were analyzed with FlowJo 10.6.1 software (TreeStar). All antibodies were diluted at a ratio of 1:200. Compensation was achieved using UltraComp beads (Thermo Fisher Scientific, 01-2222-42).

### Enzyme-linked immunosorbent assay

T cells were co-cultured with AGS cells and the supernatant was collected. Following the manufacturer’s instructions, IFN-γ (Sigma-Aldrich, RAB0222) and Granzyme B (abcam, ab235635) were detected using an ELISA kit. Samples were added to both the standard wells and sample wells, followed by incubation at 37°C. The plate was then washed. Next the biotinylated antibody working solution was added and incubated at 37°C, after which the plate was washed again. HRP-streptavidin was subsequently added and incubated at 37°C. Following another wash, TMB color developing substrate was added for incubation at 37°C. After color development, the termination solution was added. Finally, the OD450 value was measured using an ELISA reader.

### LDH cytotoxicity assay

Cytotoxicity assay was detected by LDH Cytotoxicity Assay Kit(Biyuntian Biotechnology, C0017) according to the manufacturer’s protocol. 100 μL AGS cells transfected with NALCN knockdown or control (1 × 10^5^ per well) were seeded into 96-well plates, then adding 100 μL T cell suspension (the ratio T cell and AGS cells was 1:1,5:1,10:1), cultured at 37°C for 24 and 48 hours. One hour before the predetermined detection time, LDH release reagent was added into the control hole of the maximum enzyme activity of the sample, and the mixture was blown and continued to incubate. After reaching the predetermined time, the cell culture plate was 400g and centrifuged for 5 minutes. The supernatant of each hole was 120 μL and added to the new 96-well plate. 60 μL LDH test solution was added to 96-well plate, incubated at room temperature for 30 minutes, and measure absorbance at 490 nm.

### Q300 metabolomics analysis

A GC cell sample was homogenized for 3 minutes, followed by the addition of 150 μL of methanol containing an internal standard to extract metabolites. The sample was then subjected to a second homogenization for 3 minutes and subsequently centrifuged at 18000g for 20 minutes. The supernatant was carefully transferred to a 96-well plate. All subsequent procedures were conducted on an Eppendorf epMotion Workstation (Eppendorf Inc., Humburg, Germany). Specifically, 20 μL of freshly prepared derivatization reagents was added to each well. The plate was sealed and incubated at 30°C for 60 minutes to facilitate derivatization. Following this, the sample was evaporated for 2 hours. Next, 330 μL of ice-cold 50% methanol solution was added to reconstitute the sample. The plate was stored at -20°C for 20 minutes and then centrifuged at 4000g for 30 minutes at 4°C. Finally, 135 μL of the supernatant was transferred to a new 96-well plate, with 10 μL internal standards added to each well. Serial dilutions of derivatized stock standards were added to the leftmost wells. The plate was sealed and prepared for LC-MS analysis.

### Statistical analysis

Data analysis was conducted utilizing GraphPad Prism 9.5 Software (California, USA). For normal distribution data, Student’s t-test were employed to assess differences between two groups, while one-way analysis of variance (ANOVA) was utilized for comparisons involving three or more groups. Each experiment was repeated a minimum of 3 times. Statistical significance was defined as P < 0.05.

## Results

### Low NALCN expression in GC correlates with poor prognosis

**Figure 1:**
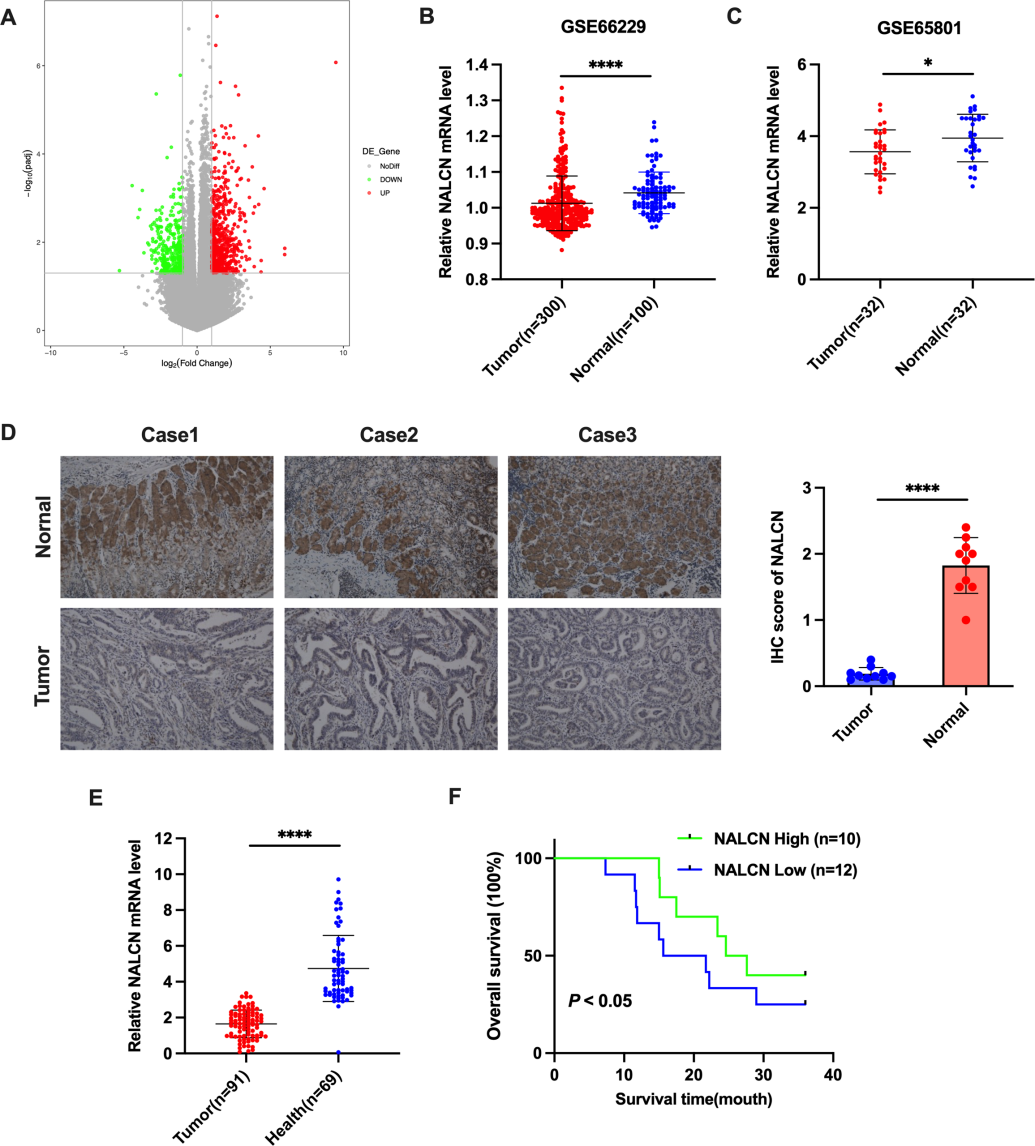
NALCN expression was downregulated in GC. **(A)** A volcano map of differentially expressed genes from 22 pairs of matched GC tumor tissues and adjacent normal tissues based on RNA-seq assay. **(B, C)** Verification of NALCN expression in GC tumor tissues in the GEO database (GSE66229 and GSE65801). **(D)** Representative image and immunochemistry intensity scoring of NALCN in tissue samples from three patients with GC (n=10). **(E)** The expression of NALCN in GC plasma (n=91) and healthy plasma (n=69). **(F)** The impact of NALCN expression on GC patients’ survival. *P<0.05, ****P<0.0001. (Scale bar = 100 μm).

In this study, we performed RNA sequencing analysis of fresh frozen tumor tissues and adjacent non-tumor tissues from 22 GC patients. We identified nine hundred and fifty-five mRNAs that were differentially expressed in GC tissues versus adjacent non-tumor tissues. Six hundred of these mRNAs were upregulated, and three hundred and fifty-five were downregulated, in GC tissues. Notably, NALCN was significantly downregulated in GC tissues (**Figure 1A**). To investigate the potential oncogenic role of NALCN in GC tissues, we analyzed its expression levels using the GEO database. Significant differences in NALCN mRNA expression were observed between tumor and non-tumor tissues in the GSE66229 and GSE65801 datasets (**Figures 1B, C**). Subsequently, IHC staining revealed lower NALCN protein expression in tumor tissue compared to adjacent normal tissues (**Figure 1D**). To further validate these findings, we examined NALCN expression in plasma samples from GC patients and healthy controls. The results demonstrated a significant reduction in NALCN expression in the plasma of GC patients relative to healthy individuals (**Figure 1E**). Importantly, Kaplan-Meier analysis indicated that decreased NALCN expression correlated with poorer overall survival (**Figure 1F**). Collectively, our findings suggest that NALCN downregulation in GC is associated with adverse prognosis and may play a role as a novel antioncogene in GC progression.

### Effect of NALCN knockdown on biological function of GC cells

**Figure 2:**
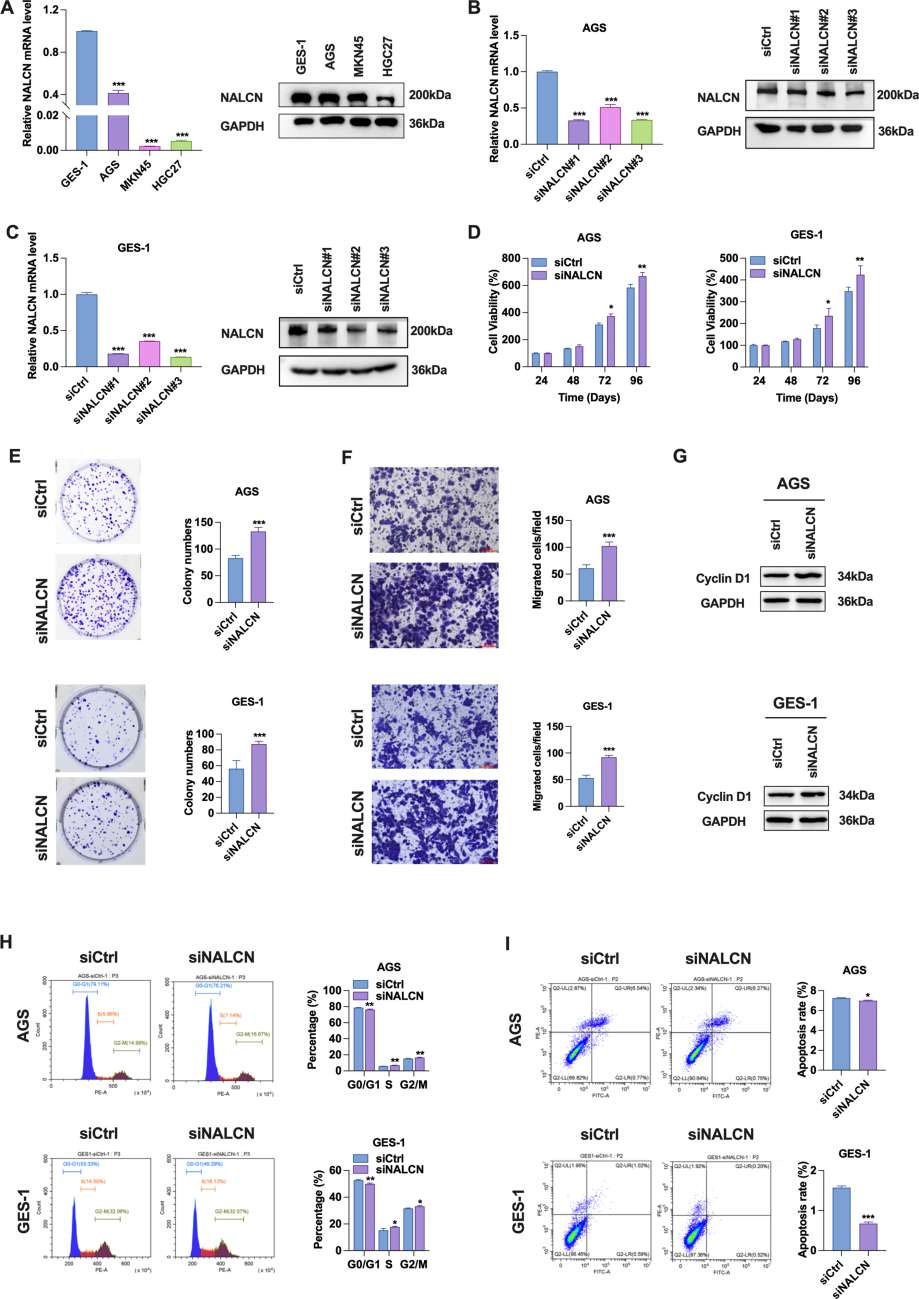
Effect of NALCN knockdown on biological function of GC cells. **(A)** The expression of NALCN was determined in three gastric cancer cell lines (AGS, MKN45 and HGC27) and normal gastric mucosal epithelial cells (GES-1) by RT-qPCR and WB. **(B, C)** RT-qPCR and WB were performed to check the relative expression of NALCN in AGS and GES-1 cells with NALCN knock down (siNALCN#1, siNALCN#2 and siNALCN#3). **(D)** CCK-8 assay showed cell proliferation after NALCN knock down in AGS and GES-1 cells. **(E)** Colony formation analysis of AGS and GES-1 colony numbers with NALCN knockdown. **(F)** The effect of NALCN knockdown on the migration of AGS and GES-1 cells was investigated using Transwell assay. **(G)** Cyclin D1 protein levels in NALCN knockdown. **(H)** Flow cytometric analysis of cell cycle in NALCN knockdown. **(I)** Flow cytometric analysis of apoptosis frequency in NALCN knockdown. *P<0.05, **P < 0.01, ***P < 0.001. (Scale bar = 100 μm).

Initially, we investigated the expression levels of NALCN mRNA and protein in GSE-1 and GC cells. The results demonstrated a significant reduction in NALCN mRNA and protein expression in GC cells (**Figure 2A**). To elucidate the role of NALCN in GC cells, we established three distinct NALCN knockdown (siNALCN#1, siNALCN#2 and siNALCN#3). NALCN expression was markedly diminished by siRNA mediated knockdown in AGS and GES-1 cells (**Figures 2B, C**). Functional assays revealed that NALCN knockdown significantly enhanced cell proliferation, colony formation and migration in both AGS and GES-1 cells (**Figures 2D–F**). Subsequently, we examined the effects of NALCN on cell cycle and apoptosis in GC cells. Our findings indicated that NALCN knockdown led to notable increase in cyclin D1 protein levels (**Figure 2G**), a decrease in the proportion of cells in G0/G1 phase, and an increase in the proportion of cells in G2/M phase (**Figure 2H**). Additionally, NALCN knockdown reduced the percentage of apoptotic cells in AGS and GES-1 cells (**Figure 2I**). Collectively, these results suggest that NALCN exerts an inhibitory effect on the biological function of GC cells.

### NALCN expression was correlated with immune infiltrate patterns of GC tumor tissue

**Figure 3:**
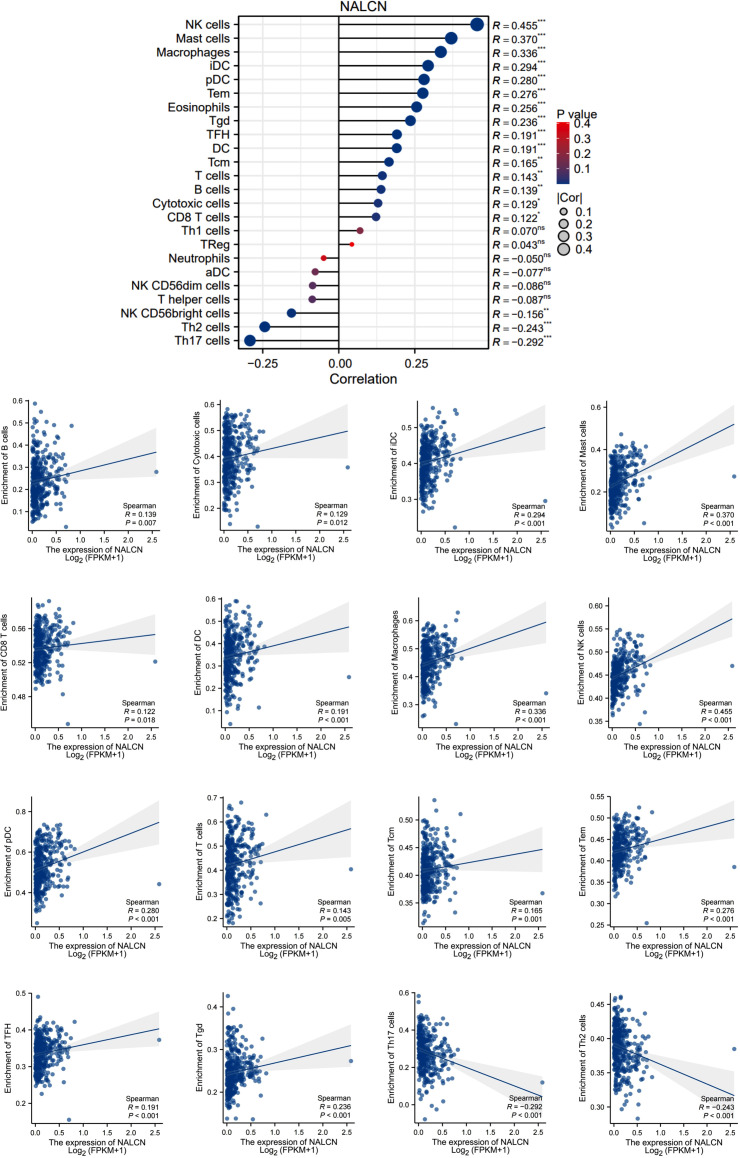
NALCN expression was correlated with immune infiltrate patterns of GC tumor tissue. *P<0.05, **P < 0.01, ***P < 0.001, ns, not significant.

We investigated the correlation between NALCN expression and the infiltration of various immune cell in GC tissue using data from the TCGA database. Our analysis revealed that NALCN expression was positively correlated with the infiltration of B cells, Cytotoxic cells, iDC cells, Mast cells, CD8^+^ T cells, DC cells, macrophages, NK cells, pDC cells, T cells, Tcm cells, Tem cells, TFH cells and Tgd cells. Conversely, the infiltration of Th17 cells and Th2 cells exhibited a negative correlation with NALCN expression. (**Figure 3**).

**Figure 4:**
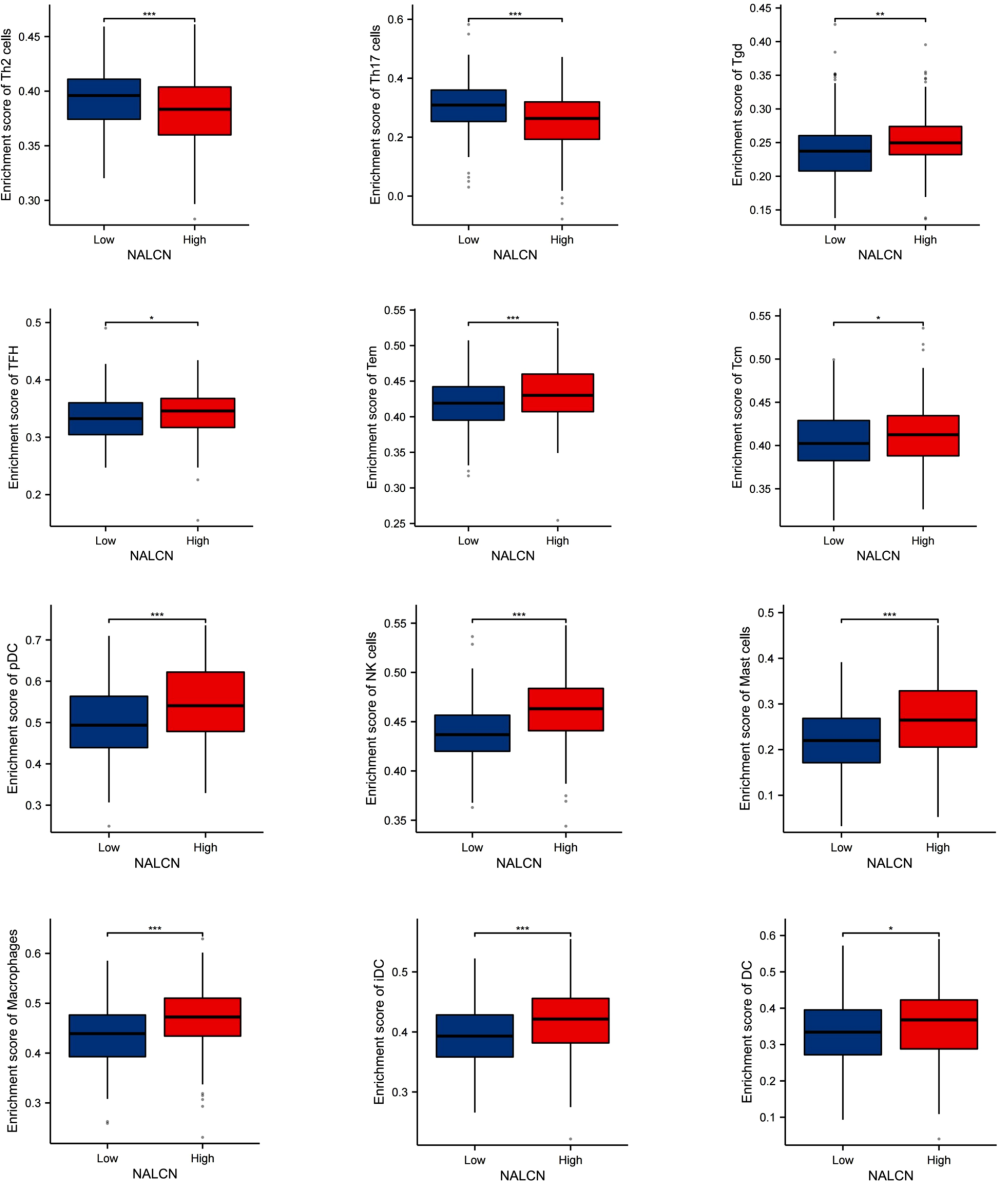
The enrichment of immune cells between the NALCN high group and NALCN low group. *P<0.05, **P < 0.01, ***P < 0.001.

**Figure 5:**
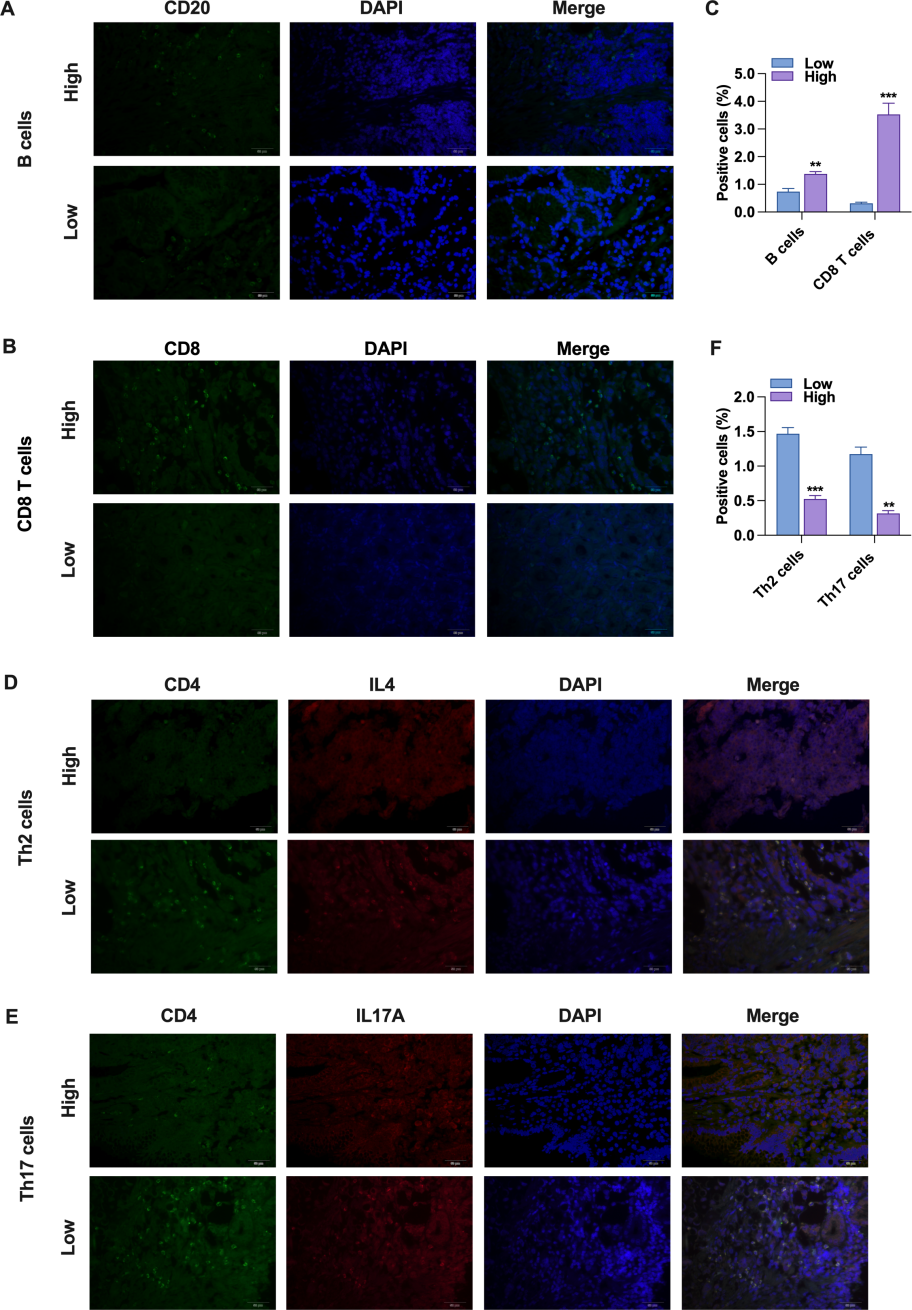
Immunofluorescence analysis in GC tumor tissue. **(A, B)** Immunofluorescence analysis was used to detect the infiltration of CD20 (green) and CD8 (green) in gastric cancer tissue sections. The nuclei were DAPI (blue) stained. **(D, E)** Immunofluorescence analysis was used to detect the infiltration of CD4 (green) were co-located with IL-4 (red) or IL-7A (red) in gastric cancer tissue sections. The nuclei were DAPI (blue) stained. **(C, F)** Percentage of positive cells.**P < 0.01, ***P < 0.001. (Scale bar = 50 μm).

To further investigate the relationship between NALCN expression and immune cell infiltration, we stratified the TCGA database into high NALCN expression and low NALCN expression groups. We then analyzed and identified the enrichment of immune cells between these two groups (**Figure 4**). Notably, both Th2 and Th17 cell populations were observed to be reduced in the high NALCN expression group. And Tgd cells, TFH cells, Tem cells, Tcm cells, pDC cells, NK cells, Mast cells, macrophages, iDC cells, and DC cells showed an elevation in the NALCN high group. Meanwhile, tissue immunofluorescence staining showed that in GC tumor tissues with high NALCN expression, there were more B cells and CD8 T cells clustered (**Figures 5A–C**), and fewer Th17 cells and Th2 cells clustered (**Figures 5D–F**). These results indicate that NALCN has close relationship with immune cells infiltration, and NALCN might be a novel immune target in cancer therapies.

### Knockdown of NALCN inhibited T cell proliferation and cytotoxic T lymphocyte activity

**Figure 6:**
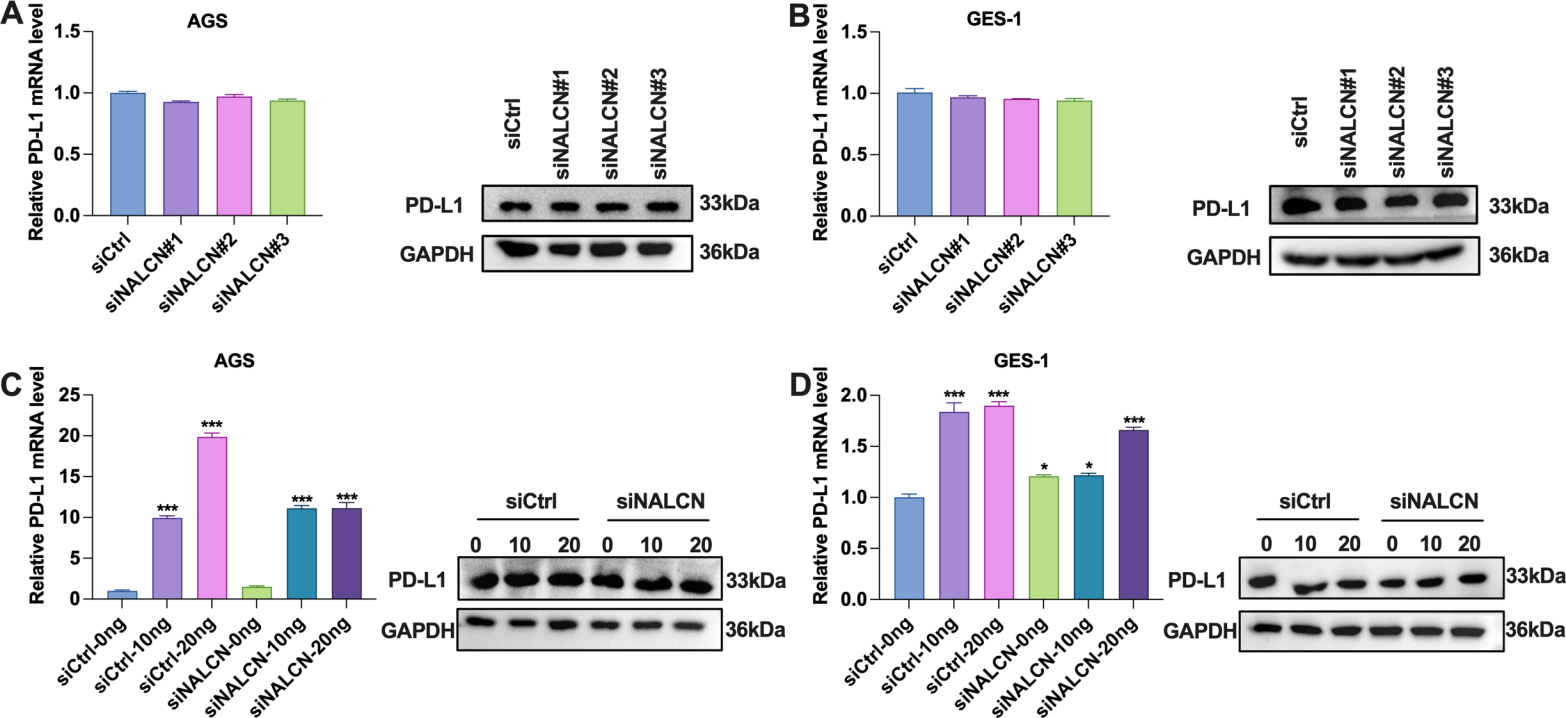
The expression level of PD-L1 in the cells. **(A, B)** RT-qPCR and WB were performed to check the relative expression of PD-L1 in AGS and GES-1 cells with NALCN knock down (siNALCN#1, siNALCN#2 and siNALCN#3). **(C, D)** RT-qPCR and WB were performed to check the relative expression of PD-L1 in AGS and GES-1 cells transfected with different concentrations of siNALCN. *P<0.05, ***P < 0.001.

To further investigate the relationship between NALCN and tumor immunity, we examined the correlation between NALCN and PD-L1 expression *in vitro*. Initially, we assessed the PD-L1 expression level in GC cells transfected with various siNALCN constructs. Our results indicated that, compared to the control group, PD-L1 expression did not significantly change following NALCN knockdown (**Figures 6A, B**). Subsequently, we evaluated PD-L1 expression in GC cells transfected with different concentrations of siNALCN. Consistent with our initial findings PD-L1 expression remained unchanged relative to the control group after NALCN knockdown (**Figures 6C, D**). These observations suggest that NALCN may influence tumor immunity through mechanisms other than altering PD-L1 expression in tumor cells, potentially modulating T cell function.

**Figure 7:**
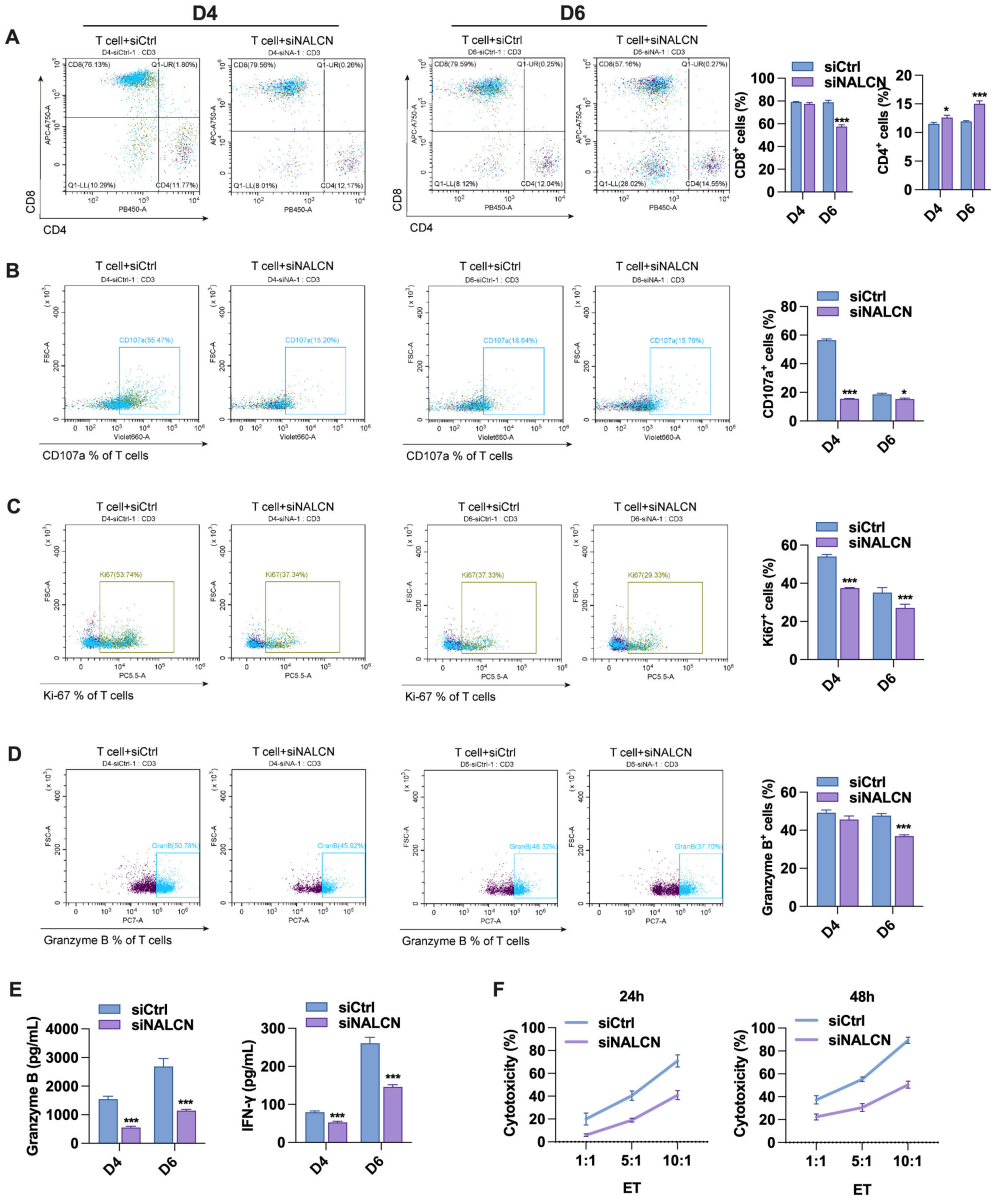
The role of NALCN in regulating T cell function (GC cells co-cultured with T cells transfected with NALCN knockdown or control). **(A–D)** The expression levels of CD4^+^, CD8^+^, CD107a, Ki67 and GZMB were detected by flow cytometry. **(E)** The secretion of granzyme B and IFN-γ in the medium. **(F)** The killing function of T cells on tumor cells was examined by LDH cytotoxicity assay. *P<0.05, ***P < 0.001.

To test our hypothesis, we investigated the role of NALCN in regulating T cell function through two distinct approaches. First, we co-cultured GC cells transfected with NALCN knockdown or control vectors with T cells for 4 and 6 days. Flow cytometry analysis revealed that the proportion of CD8^+^T cells decreased while the proportion of CD4^+^T cells increased after co-culture (**Figure 7A**), suggesting that NALCN knockdown significantly impaired the antitumor activity of CD8^+^T cells. Consistent with these results, co-culture of GC cells with NALCN knockdown resulted in reduced expression levels of CD107a, Ki67 and granzyme B in T cells (**Figures 7B–D**). Additionally, the secretion of granzyme B and IFN-γ in the co-culture medium was also diminished (**Figure 7E**). In a subsequent experiment, we co-cultured these cells with T cells for 24 and 48 hours. We observed that tumor cells with NALCN knockdown exhibited greater resistance to T cell-mediated cytotoxicity compared to control cells (**Figure 7F**).

**Figure 8:**
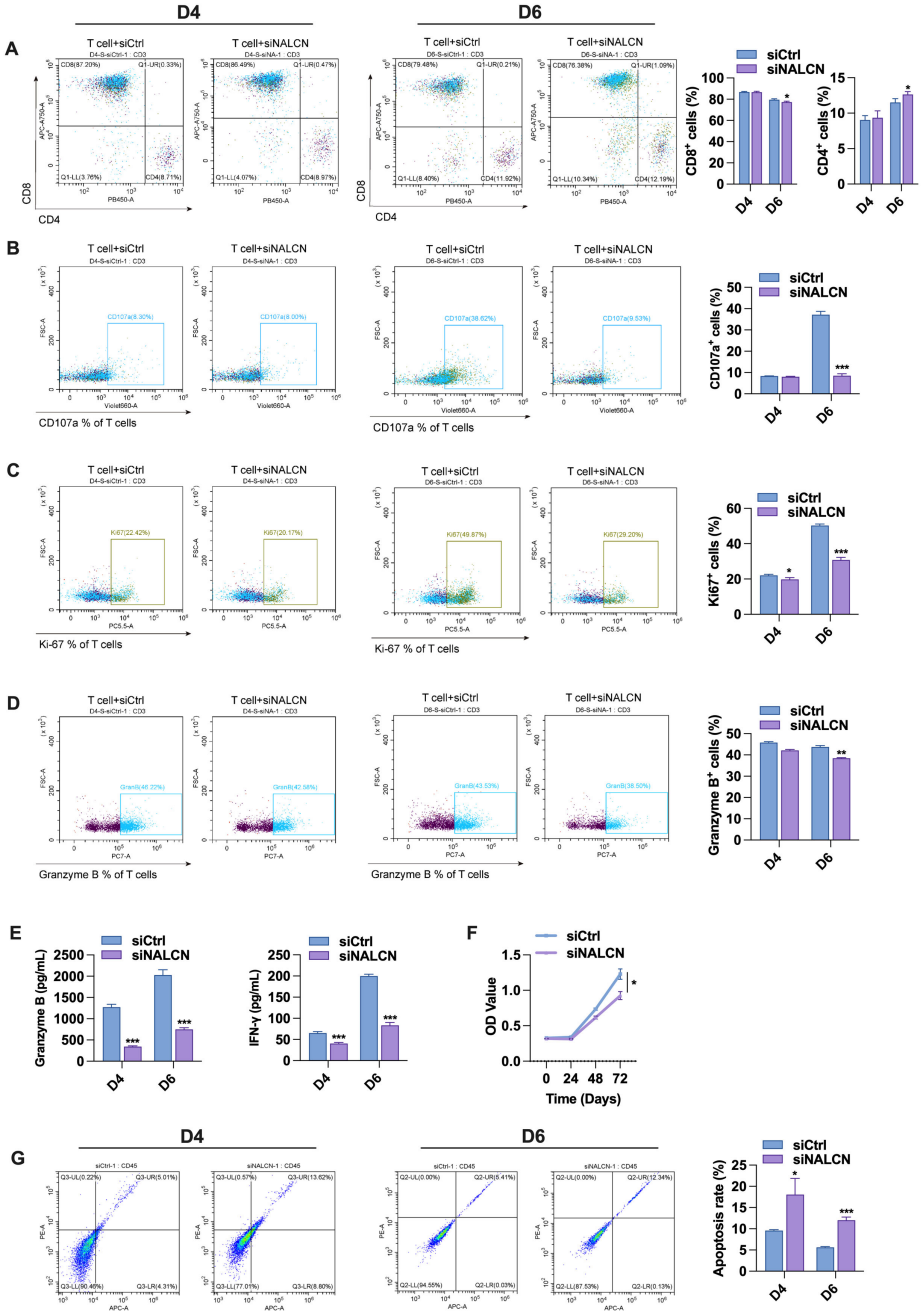
Effect of AGS cell supernatant transfected with control and NALCN knockdown on T cell function. **(A–D)** The expression levels of CD4^+^, CD8^+^, CD107a, Ki67 and GZMB were detected by flow cytometry. **(E)** The secretion of granzyme B and IFN-γ in the medium. **(F)** T cell proliferation by CCK-8 assay. **(G)** The frequency of T cell apoptosis by flow cytometry. *P<0.05, **P < 0.01, ***P < 0.001.

Next, we collected the culture supernatant from GC cells transfected with either control vectors or NALCN knockdown. The supernatants were used to stimulate T cells for 4 and 6 days, respectively. Flow cytometry was employed to assess the expression levels of CD4^+^, CD8^+^, CD107a, Ki67 and GZMB in the stimulated T cells. The results showed that the supernatant from NALCN knockdown cells led to a decrease in CD8^+^ T cell expression and an increase in CD4^+^ T cell expression (**Figure 8A**), suggesting that NALCN knockdown significantly impaired the antitumor activity of CD8^+^ T cell. Consistent with the results of co-culture, the expression of CD107a, Ki67 and granzyme B in T cells was decreased after stimulation with the supernatant from NALCN knockdown GC cells (**Figures 8B–D**). And the secretion of granzyme B and IFN-γ in the co-culture medium was also diminished (**Figure 8E**). Next, we evaluated T cell function using CCK8 assays and apoptosis analysis. The results showed that, compared to control cells, the supernatant from NALCN knockdown cells inhibited T cell proliferation (**Figure 8F**) and promoted T cell apoptosis (**Figure 8G**). It is suggested that NALCN knockdown may suppress anti-tumor immunity by inhibiting T cell proliferation and promoting tumor development.

### Cellular glucose and glutamine metabolism disorders after NALCN knockdown

**Figure 9:**
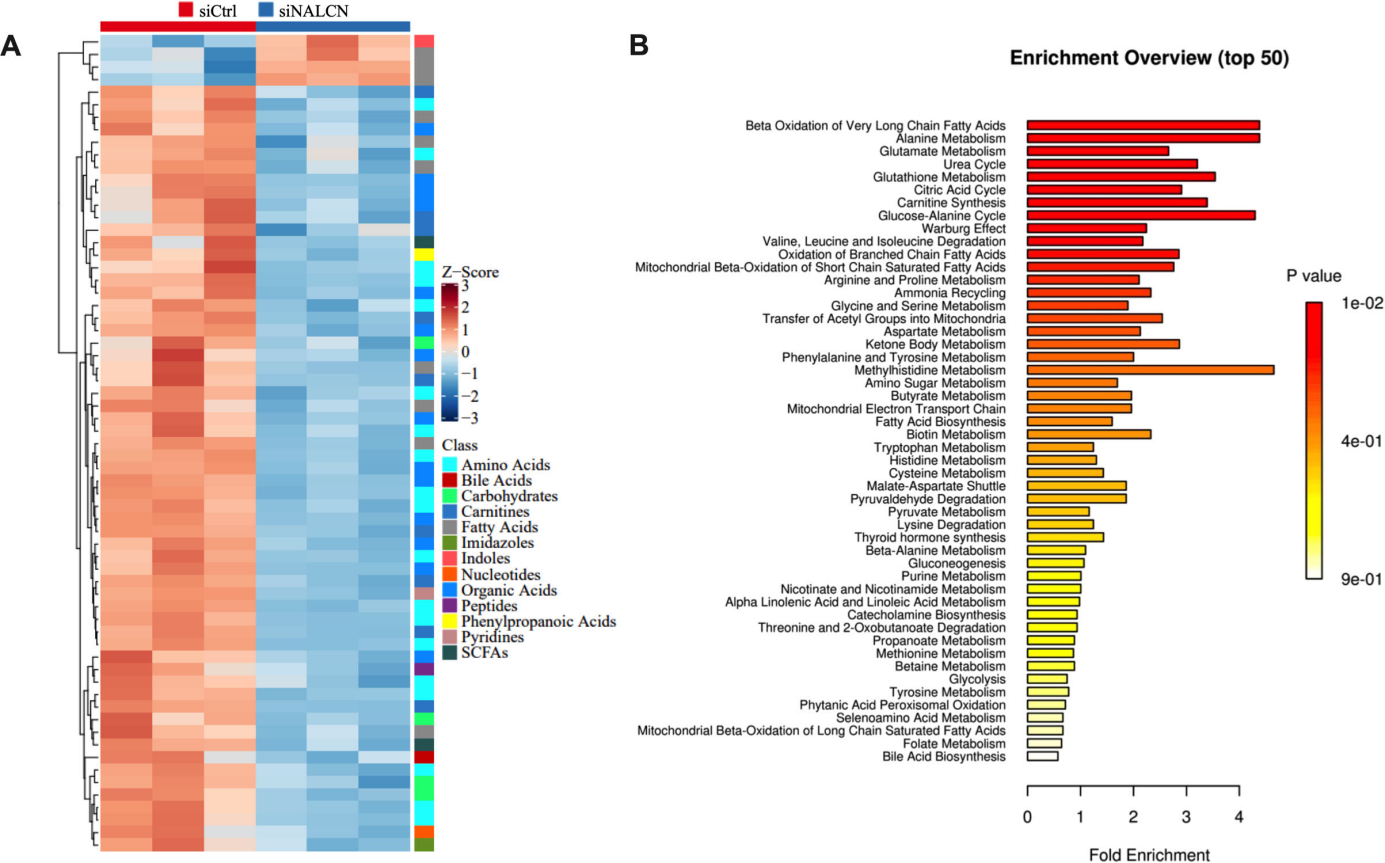
The heatmap and enriched metabolic pathway analysis of significantly differential metabolites. **(A)** Heatmap of cell levels of significantly differential metabolites between transfected with NALCN knockdown and control GC cells. **(B)** Enriched metabolic pathway analysis of transfected with NALCN knockdown and control GC cells.

Finally, GC cells transfection control and NALCN knockdown were collected for Q300 metabolomics analysis. We carried out cluster analysis on the significantly differential metabolites between transfected with NALCN knockdown and control GC cells (**Figure 9A**). To gain insights into the altered metabolites in NALCN knockdown GC, we conducted metabolite sets enrichment over view analysis. Among NALCN knockdown GC cell, the top differential pathways compared to control were beta oxidation of very long chain fatty acids, alanine metabolism, glutamate metabolism and glucose-alanine cycle (**Figure 9B**). It is suggested that NALCN may promote glucose and glutamine uptake by Na^+^ dependent transport system.

## Discussion

NALCN has traditionally been considered a single ion channel located on the plasma membrane and classified within the four-domain ion channel superfamily (11). The NALCN protein contains 1738 amino acids, constitutes a complete membrane ion channel complex. In Homo sapiens, NALCN is encoded by a single gene (21, 22). This protein plays an essential role in maintaining cellular resting and excitable membrane potentials and is implicated in multiple processes, including sensitivity to volatile anesthetics and motor behavior (15, 23, 24). Moreover, the NALCN gene may serve as a susceptibility locus for several diseases, such as alcoholism, Alzheimer’s disease, autism, bipolar disorder, cardiovascular conditions, epilepsy and schizophrenia (24). Research indicates that NALCN expression is observed in certain cancers, including glioblastoma, non-small cell lung cancer (NSCLC), pancreatic cancer, small cell lung cancer (SCLC), and tumor-derived endothelial cells (24–26). Furthermore, He et al. demonstrated that in TCGA datasets, the expression level of NALCN was significantly downregulated in bladder urothelial carcinoma (BLCA), breast invasive carcinoma (BRCA), cervical squamous cell carcinoma and endocervical adenocarcinoma (CESC) and colon adenocarcinoma (COAD), but upregulated in cholangiocarcinoma (CHOL), pheochromocytoma and paraganglioma (PCPG), stomach adenocarcinoma (STAD). Their findings indicate that NALCN expression exhibits tissue specific and is aberrantly regulated in multiple cancer types, indicating its potential as a crucial biomarker for cancer diagnosis (27). More recently, Richard J. Gilbertson’s team also reported that NALCN regulates the transfer of epithelial cells to distant tissues, and loss of NALCN function promotes cancer metastasis (19).

In this study, we analyzed data from the GEO database and the mRNA expression levels in GC patient tissues collected by our research team. Our findings revealed a significant downregulation of NALCN expression in GC tissues compared to normal tissues. Consistently, at the protein level, NALCN expression was also markedly reduced in GC tissues relative to normal tissues. These results suggest that NALCN may function as a potential tumor suppressor in GC. However, our findings were inconsistent between the expression trends of NALCN in GC tissues from our study and TCGA database reported by He et al. (27). Several factors may contribute to this discrepancy. Firstly, NALCN expression may vary across different pathological types of GC, as our tissue samples included not only STAD but also other subtypes. Secondly, NALCN expression could be tissue specific, which necessitates further investigation with an expanded sample size to validate this hypothesis. Subsequently, we performed immunofluorescence staining on GC patient tissues to characterize the expression levels of NALCN and investigate its potential correlation with immune cell infiltration. The results demonstrated that elevated NALCN expression was associated with increased immune cell aggregation and higher expression of immune cell markers. These findings indicate that NALCN is likely to play a role in the regulation of multiple immune cells and may possess potential prognostic significance in cancer immune therapies.

GC is among the most prevalent types of malignant tumors. Despite notable advancements in early diagnosis and clinical treatment, the prognosis for patients with GC remains suboptimal (28). In recent years, the emergence of immunotherapy has presented novel and promising opportunities for managing these refractory tumors (29). TIME plays a pivotal role in tumor development and response to immunotherapy. In the new era of immunotherapy, it is crucial to identify specific biomarkers associated with TIME to predict clinical outcomes and stratify patients for selecting the most effective therapeutic strategies. During tumor progression, the interaction between TIME and tumor cells can lead to tumor immune tolerance, thereby influencing the anti-tumor immune response (30).

TIME comprises tumor cells, immune cells, cytokines, and other components. The interaction among these elements determines the direction of anti-tumor immunity, which can be is categorized into anti-tumor and pro-tumor. Anti-tumor immune cells primarily include effector T cells (such as cytotoxic CD8^+^ T cells and effector CD4^+^ T cells), natural killer cells (NK), dendritic cells (DC). Conversely, tumor-promoting immune cells mainly consist of regulatory T cells (Tregs), myeloid-derived suppressor cells (MDSCs), M2-polarized macrophages, etc. Additionally, metabolic and biochemical factors significantly influence the function of immune cell (31).

An increasing body of research demonstrates that TIME is intricately linked to the clinical efficacy of immunotherapy. D’Alterio et al. reported that modulating CXCR4 within the TIME enhanced T cell infiltration, thereby improving the effectiveness of anti-PD-1 therapy (32). A recent study revealed that concurrent inhibition of PD-L1, CXCR1/2, and TGF-β significantly augmented T-cell infiltration and activation in the tumor microenvironment, consequently enhancing the antitumor activity of ICI (33). Therefore, a comprehensive understanding of the molecular characteristics and immune status of TIME can facilitate the development of personalized immunotherapies for different GC patients. Currently, the availability of high-throughput sequencing data and large-scale immunization databases enables the identification of novel and reliable immune-related biomarker. Previous studies have utilized algorithms based on gene expression to develop immune signatures that predict clinical outcomes in patients with hepatocellular carcinoma or lung cancer (34, 35). However, there remains a lack of biomarkers capable of systematically evaluating TIME and predicting prognosis or immunotherapy response in GC patients.

To summarize, this study represents the pioneering investigation into the relationship between NALCN and various types of immune cells through immunofluorescence staining. By leveraging publicly available databases, we confirmed the significant role of NALCN in the GC immune microenvironment. Immunofluorescence staining revealed that CD8^+^ T cells and B cells exhibited higher infiltration levels in samples with elevated NALCN expression, whereas lower NALCN expression was associated with reduced immune cell infiltration. These findings suggest that NALCN may play a crucial role in the regulating immune cell infiltration, thereby influencing the tumor microenvironment and immune response, potentially contributing to immune escape. Consequently, NALCN’s influence on the tumor microenvironment may present a potential therapeutic target. However, further research is required to elucidate the precise mechanism by which NALCN affects GC immune infiltration. To explore this, we conducted co-culture experiments involving NALCN knockdown or control GC cells with T cells. The results indicated that NALCN knockdown significantly diminished the antitumor activity of CD8^+^ T cells, reduced the granzyme B and IFN-γ secretion in T cells, inhibited T cell proliferation (**Figures 5B–D**), and promoted T cell apoptosis. This suggests that NALCN knockdown may suppress anti-tumor immunity by inhibiting T cell function and promoting tumor progression. Low NALCN expression is associated with altered tumor immunity and poor prognosis in GC patients.

However, there are several limitations. Firstly, bioinformatics analysis and patient tissue samples were utilized solely to verify the relationship between NALCN and immune cell infiltration. Considering the complexity of the TIME, employing animal models could provide a more comprehensive understanding of NALCN’s role in GC immunity. The primary objective of this study was to investigate the correlation between NALCN expression and immune infiltration and its impact on the prognosis of GC patients. This research contributes to our understanding of the pivotal role of NALCN in human tumors, including GC.

## Conclusions

Our current study provided compelling evidence that elevated expression of NALCN promotes immune cell aggregation in GC and can serve as a potential biomarker for immune infiltration. This investigation elucidated several possible roles of NALCN in regulating immune cell infiltration with GC tissues. Nevertheless, further research is warranted to explore the molecular mechanism underlying NALCN’s role in tumorigenesis and its clinical application prospect.

## Data Availability

Publicly available datasets were analyzed in this study. This data can be found here: TCGA: TCGA-STAD; GEO: GSE66229 and GSE65801.
