# Supplementary figures and images for: NALCN expression is down-regulated and associated with immune infiltration in gastric cancer
Source: Front Immunol. 2025 Feb 12;16:1512107. doi: 10.3389/fimmu.2025.1512107 (PMC11860897; doi:10.3389/fimmu.2025.1512107)

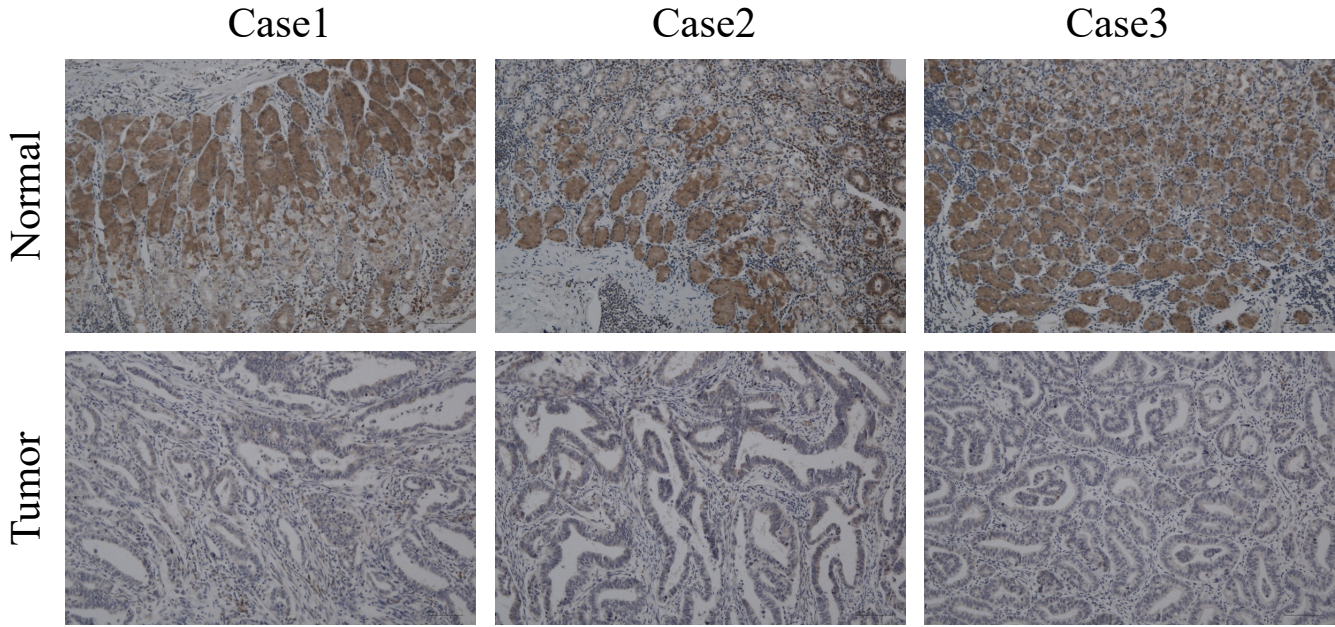

2A

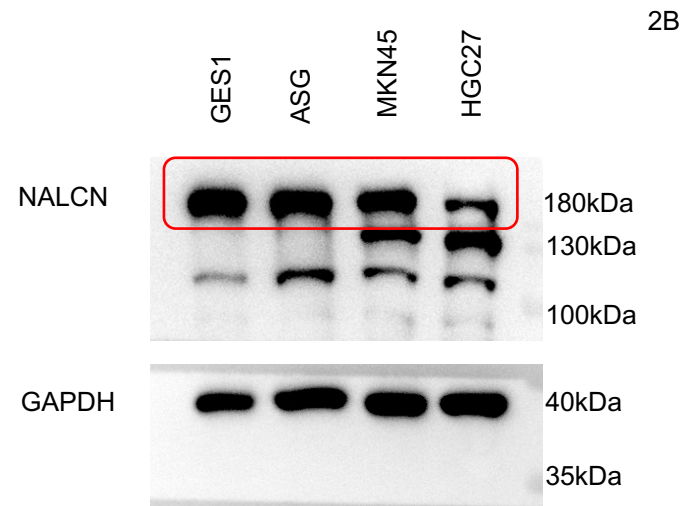

2B

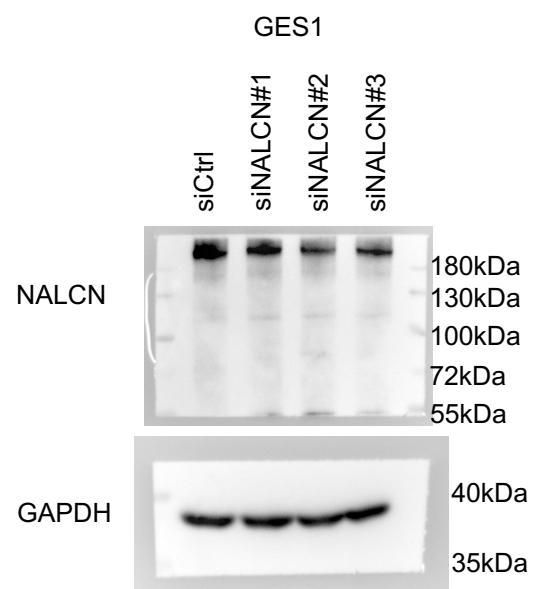

2C

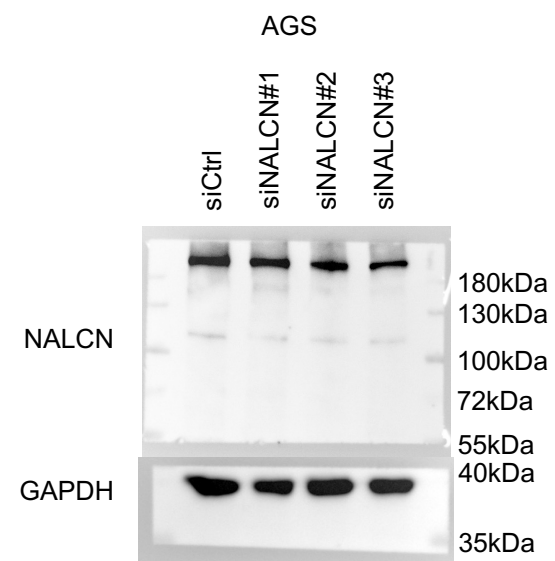

2E

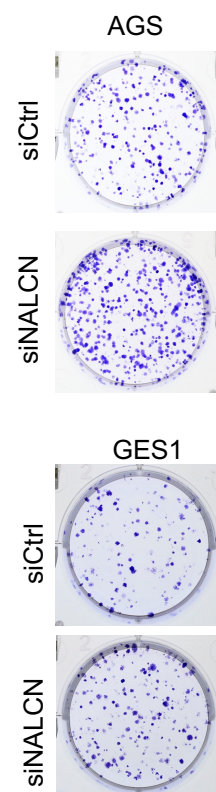

2F

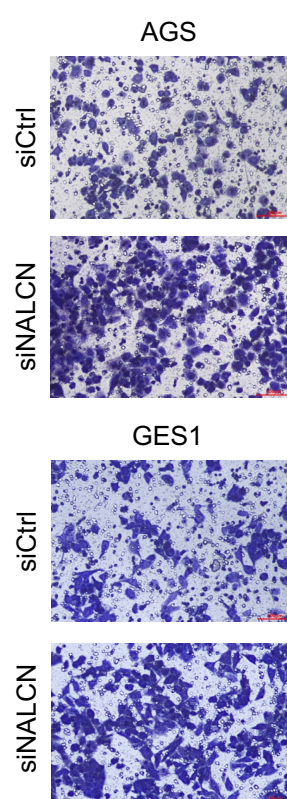

5A

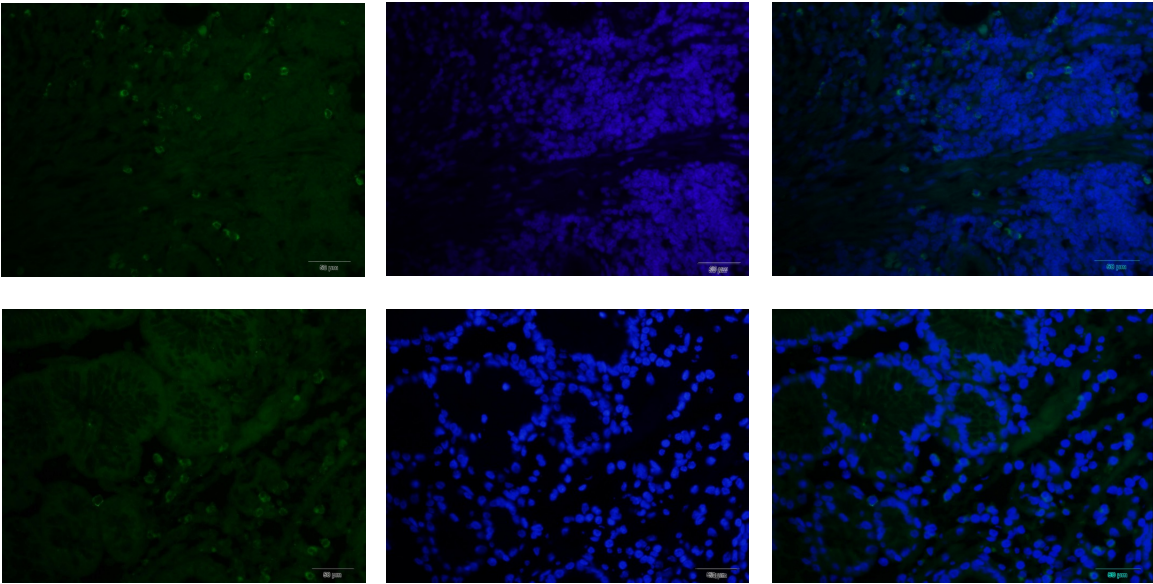

5B

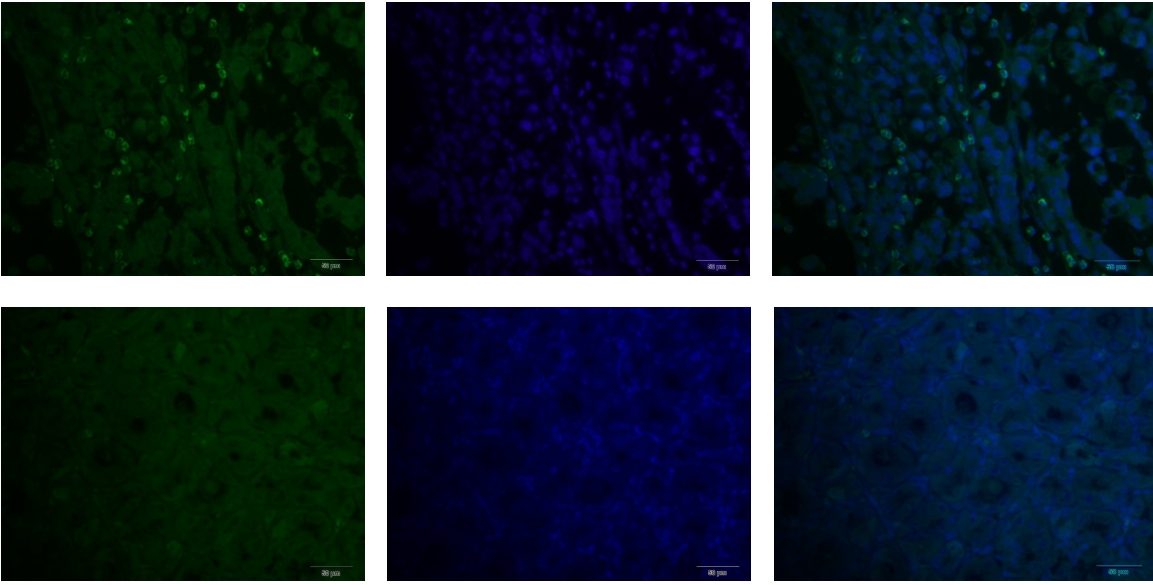

5C

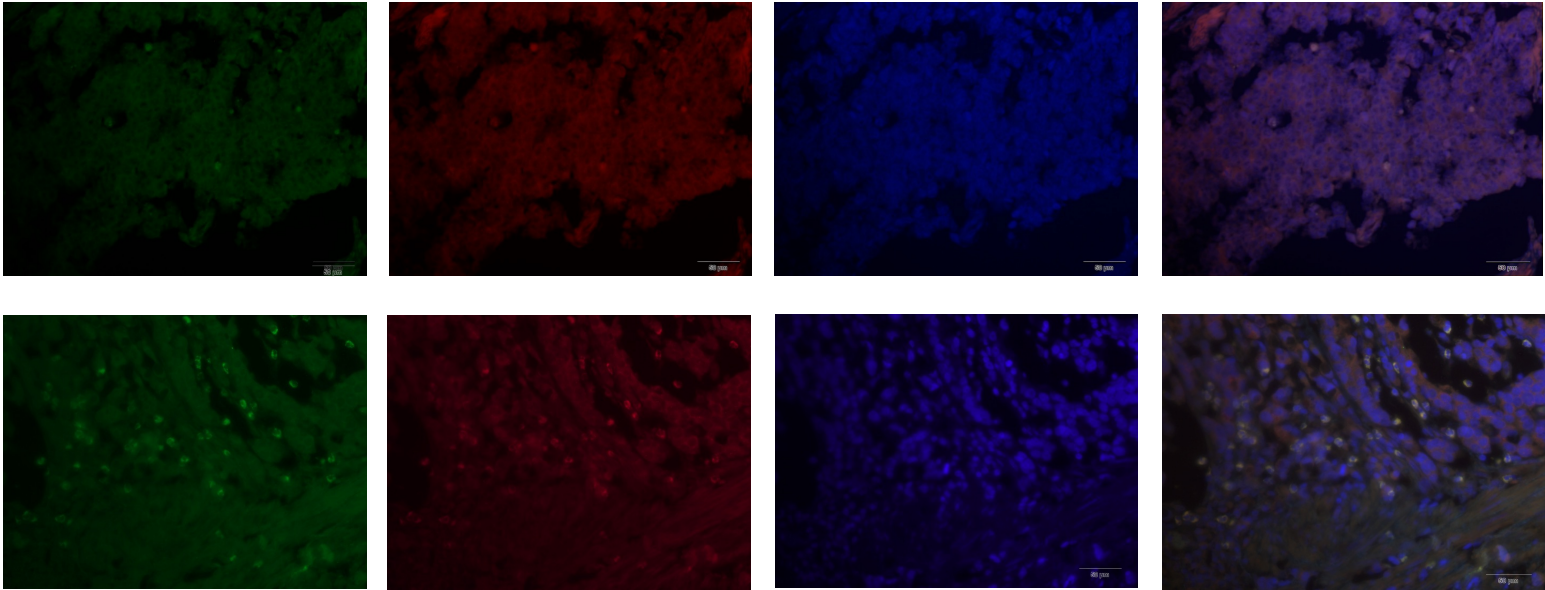

5D

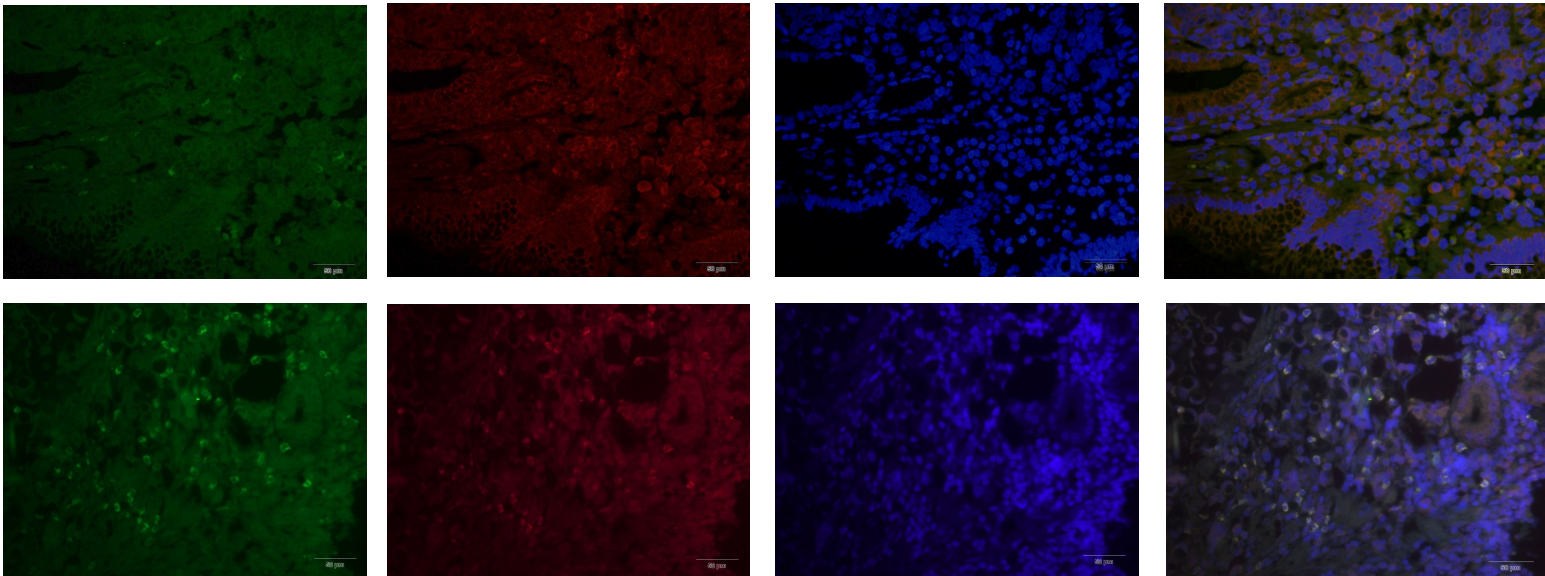

6A

AGS

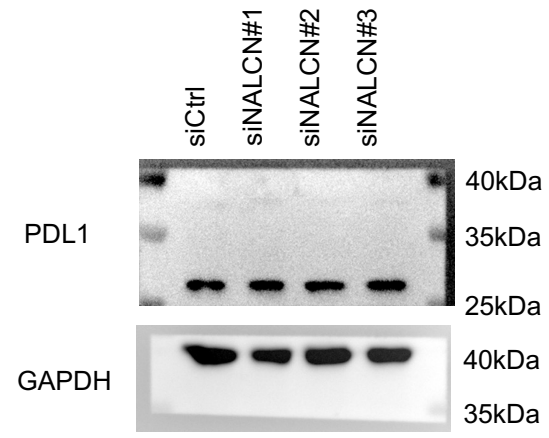

6B

GES1

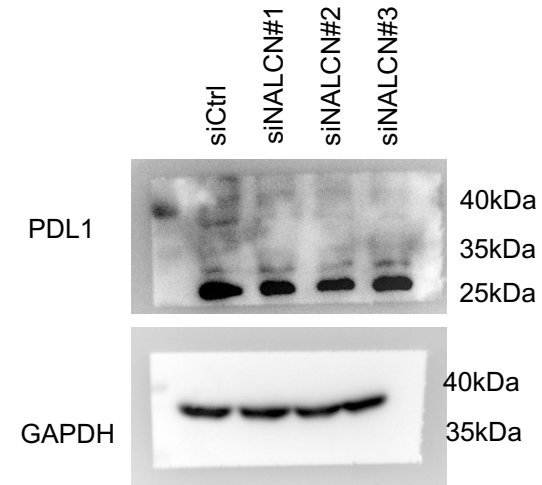

6C

siCtrl siNALCN

0 10 20 0 10 20

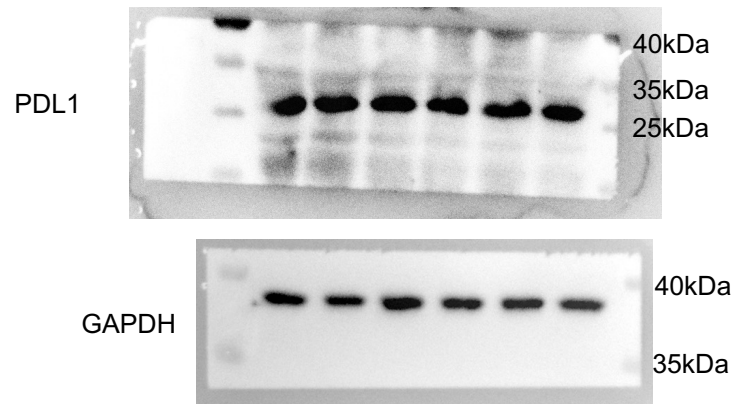

6D

siCtrl siNALCN

0 10 20 0 10 20

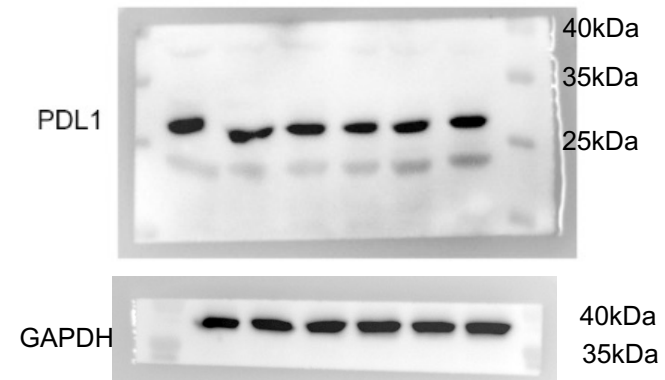

Supplement: Supplementary file 1 [file DataSheet1.pdf]
